# Home-based training technology for persons with dementia: a qualitative study of barriers and facilitators for mobility-based training at home

**DOI:** 10.1186/s12877-022-03505-6

**Published:** 2022-10-14

**Authors:** Eva Ladekjær Larsen, Frans Boch Waldorff, Helle Ploug Hansen, Karen la Cour

**Affiliations:** 1 Department of Clinical Medicine, Aarhus University, Aarhus, Denmark and Horsens Regional Hospital, Horsens, Denmark; 2 Department of Public Health, Section of General Practice, Copenhagen University, Copenhagen, Denmark; 3 Department of Public Health, Research Unit of General Practice, University of Southern, Odense, Denmark; 4 Department of Public Health, Research Unit of User Perspectives & Community-Based Intervention, University of Southern, Odense, Denmark

**Keywords:** Motion-based technology, Dementia, Qualitative studies, Rehabilitation, Physical training

## Abstract

**Background:**

Physical training is increasingly used in rehabilitation for older people with dementia and several studies have documented positive results. Currently, welfare nations promote motion-based technology (MBT) at home to replace group training in various rehabilitation interventions. Research on the use of MBT by people with dementia is sparse. Therefore, this study explores how people with mild dementia and their relatives experience home-based MBT training in an intervention facilitated by a Danish municipality.

**Methods:**

The study is part of a feasibility study and builds on participant observation and interviews with people with dementia (*n* = 4), their relatives (*n* = 4), and health care workers (*n* = 3) engaged in the project.

**Results:**

Participants compared MBT training to group training and found that MBT was not a satisfactory replacement for group training. Some participants used and enjoyed MBT daily while others were challenged by the technology, the placement of the device, or motivation to independently complete the training program.

**Conclusion:**

MBT is possibly best considered as a supplement to group training, suitable for individuals able to use it in daily life.

**Supplementary Information:**

The online version contains supplementary material available at 10.1186/s12877-022-03505-6.

## Background

People with dementia are known to be less physically active compared to healthy people of the same age, which may affect quality of life and ability to maintain independence and autonomy in daily life [1]. Loss of autonomy and increased dependency are key elements experienced by people with dementia. Research has shown that people diagnosed with mild to moderate dementia who experience decreased autonomy have a diminished sense of personal dignity and well-being and increased emotional distress [2]. A central element in sustaining personal dignity is to engage in meaningful activities and social relationships [3]. Hence, group physical training can be an effective rehabilitation strategy for older individuals with dementia, resulting in positive effects [4–8]. Persons with dementia may find great pleasure and joy in participating in exercise programs [9]; exercise seems to improve self-efficacy [10] and may be seen as a means to maintain selfhood [11]. Most studies on exercise and dementia have explored physical training in groups and in institutionalized settings, although existing research suggests that individual home-based training is feasible for community-dwelling adults with mild cognitive impairment [12, 13]. However, results are inconsistent and indicate a need for further research [14].

Home-based training is a particularly relevant approach considering that physical training using motion-based technology (MBT) at home is increasingly used in therapeutic interventions [15]. MBT is characterised by interaction between physical action and technology, also referred to as 'exergames', more commonly seen outside clinical settings in sports, exercise or dance-based video games for various video game platforms [16].

The use of MBT and other technologies in the healthcare sector may be seen as part of a growing tendency to shift the provision of healthcare from institutional settings to the home [17–20]. As part of this trend, people with dementia are often expected to live at home and manage daily life despite symptoms that may have previously required hospitalization or institutionalization [21].

Home-based training using MBT for persons with dementia is a relatively new field and has already indicated potential to improve cognitive and physical functions [15, 22]. The sparse number of studies in this area may be due to dementia-related apathy resulting in lack of initiative and motivation, and difficulties following a training program [23]. Adherence to a home-based training program using MBT may be more difficult for a person with dementia, considering the cognitive demands of learning new technology.

This study reports on an MBT intervention facilitated by a Danish municipality and a university based research team. The municipality offers rehabilitation to individuals with dementia, consisting of MBT training and social and psychological support. The intervention also included support offered to relatives of the individuals with dementia. The efficacy and feasibility of the present MBT intervention is reported elsewhere [24]. In summary, the efficacy and feasibility study tested whether MBT training at home is feasible and whether it improves physical and cognitive functions and quality of life. MBT training at home showed a tendency to stabilize physical and cognitive functioning: 52% of the participants trained with MBT at home, and among them, half had high adherence to the MBT training activity. However, the results also revealed a tendency of declining quality of life with MBT implementation, for both the participants and their caregivers. These results call for further research and in this study we explore how people with dementia and their relatives experience home-based MBT. Specifically, we are interested in identifying the barriers and facilitators to the use of MBT at home and whether and how it increases quality of life.

## Methods

### Study design

People with dementia often have difficulties with auditory and visual language processing, conducting coherent conversations, and recall and abstraction. Interviewing as a standalone research method therefore has limitations [25]. The present study thus builds on multiple qualitative methods, combining participant observations, semi-structured individual interviews, focus group interviews, and informal interviews during participant observations. This combined methodological approach allows for observation of actions and immediate responses, the comparison of behavior to self-reported data, and the combination of perspectives from various data sources, and is a useful approach for people with dementia who may have diminished ability in self-expression [26].

### The intervention

The MBT intervention was based on an online administrative system developed by Welfare Denmark. The MBT instructor (physiotherapist) sat up a training program based on each participant's physical capabilities. The training program was installed on a device used in the participant's home, consisting of a touch screen, a Microsoft Kinect camera, and a modem. The device was placed at a 1.5 × 3 m' distance from the person performing the exercise and protected from sharp light. The program guided the participant via on-screen text, audio and video. The camera registered the participant's movements and participants received feedback from the device if the exercises were performed incorrectly. The device's data, consisting of performance quality of the training, time per exercise, and training frequency, was accessible by the managing physiotherapist. The difficulty level of the training program was adjusted according to the needs of the participant.

**Fig. 1:**
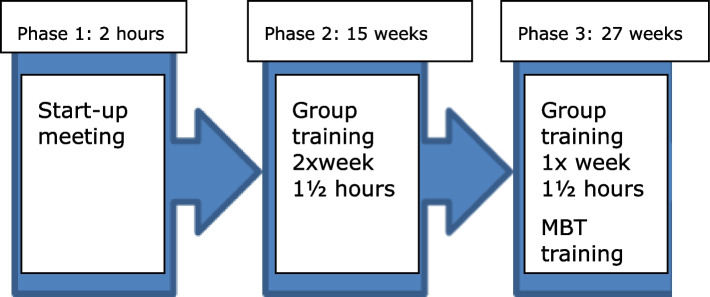
Intervention flow

The intervention flow is illustrated in Fig. 1. Phase 1 consisted of a start-up meeting in which the participant, their relative and a training instructor discussed the participant’s health, social life, participation in domestic tasks, physical activities and support needs. Beforehand, a dementia consultant had visited their home to identify resources and challenges in daily life. In phase 2, physical training facilitated by an instructor (physiotherapist) was initiated and took place in smaller groups of 4–8 participants at a local health care center. The exercises were a combination of balance, coordination, and strength exercises and cardio workout, adjusted to the level of participants' strength, flexibility, and endurance. In phase 3, the group training was reduced to once a week and participants were encouraged to train twice a week at home using the MBT.

### Participants

The inclusion criteria for participating in the intervention was as follows: a mild to moderate dementia diagnosis from a memory clinic, a Mini-Mental State Examination (MMSE) score above 18, 50 years of age or older, living at home, contact with a relative on a daily basis, and capable of expressing experiences; also, the participant’s relative had to consent to participation. Persons with other serious physical or psychiatric illness, including severe sight or hearing disabilities, were excluded. A total of 23 persons with dementia participated in the intervention.

Participants included in the present qualitative study were involved in phase 3 of the intervention. During participant-observation in a group training session and through the assistance of the training instructor, seven participants were invited to participate in the qualitative study. Afterwards, their relatives were contacted and invited. Four persons with dementia and their relatives accepted to participate in semi-structured interviews (*n* = 8). The four participants were 67 to 82 years old, three males and one female; two were diagnosed with Alzheimer’s and two with unspecified dementia. Participants were diagnosed 1–3 years prior to inclusion. The relatives were between 50 and 70 years; two were working and two were retired. All relatives were female; three spouses and one daughter. The three project workers included a female training instructor, a female dementia consultant and a male MBT instructor. The perspectives of the three project workers were included as they were key to understanding the potential barriers and facilitators faced by persons with dementia during the intervention.

### Data collection

At first a focus group interview was conducted with the three project workers. The purpose of this interview was to gain knowledge of their experiences with MBT and to identify potential themes to be discussed and observed in the study. The interview followed an interview guide containing the topics: people with dementia and physical training in general, at the health care centre, and at home; interactive communication technology (ICT) and people with dementia; the role and needs of relatives in the intervention; and potential changes to future MBT interventions.

Participant observations were conducted in three different settings. First, two start-up meetings and tests (each 2.5 h in duration) were observed at the local health care centre. Participants in these observations were the person with dementia, their participating relatives, the training instructor and the dementia consultant. At this meeting the intervention was introduced and cognitive function was measured by MMSE and Neuropsychiatric Inventory-Questionnaire (NPI-Q); and quality of life was measured by European Quality of Life 5 dimensions questionnaire (EQOL5). In addition, an interview regarding daily activities performed, and physical tests (sit to stand, timed up-and-go, 6-min walk test, and 10-m dual-task walking test) performed. These observations provided data on the abilities of the persons with dementia and potential challenges related to physical training and the use of ICT.

Second, the first author participated in two group training sessions followed by coffee and informal talks (each 2.5 h in duration) held at the local health centre. Before each training session, participants and their relatives met in the hallway of the health center for friendly conversation. While participants completed the training session, the relatives continued their conversations, sharing experiences of their relationships with the persons with dementia. An instructor guided the exercises performed individually, in the group and/or in pairs. Participants were familiar with each other and the various exercises, and the atmosphere was characterized by joy as the participants made jokes, teased, and competed. Participant observation allowed opportunities to meet the participants and to explore the interactions among the participants and between the training instructor and the participants.

Third, training sessions at home were followed to observe how participants were interacting with the technology. The interview participants were asked to perform a MBT session immediately before the interview. During these MBT training sessions, attention was paid to the placement of the device, how to turn it on and off, performing the concrete exercises, and responses to the device's feedback.

Informal conversations that arose during participant observations provided information regarding the physical training in context, whether at home or at the health care center, and such information was also used to inform the semi-structured interview guide.

Interviews were conducted in the participants' homes to support participants by being in a familiar and safe environment [27]. Interview questions were structured to elicit concrete statements, use familiar terminology, and to continuously validate the meaningfulness of their experiences. The interviews followed an interview guide and contained topics including physical training at home and at the local health care center, previous experiences with physical training, use of MBT, previous experiences with ICT, and other activities in daily life (see also supplementary file: Interview Guide). Additionally, relatives were invited to share their perspectives on MBT use and the influence it had on their relationship to the individual with dementia and relatives' wellbeing. In two cases, participants and their relatives were interviewed together, whereas the other two were interviewed separately from their relatives. The decision regarding joint or solo interviews was based on participant preference. All interviews were transcribed verbatim.

### Data analysis

The data comprised fieldnotes from participant observations and transcribed interviews, which were organised based on systematic text condensation (STC), which proceeded through four steps [28]. First, the transcriptions were carefully read with an open mind to identify preliminary themes and to get an overall impression of the contents. Second, text fragments related to the research questions were identified and labelled with codes, or meaning units. In this process, the codes were then decontextualized for comparison across data sources. Third, meaning units were condensed into an abstract format representing the meaning units across the data. Fourth, the condensed meaning units were transformed into descriptions and concepts based on the research questions. The organization of the data is illustrated in Table 1.

**Table 1:** Data analysis

Step 1: From raw data to themes	Step 2: From themes to codes (meaning units)	Step 3: From codes to meanings (condensation)	Step 4: From condensation to description (Illustrated in the results section)
-Activities in daily life -Changes in daily life -Previous experiences with physical activity -Previous experiences with ICT -Dementia and taboo -Dementia and being open -Frequency of the use of the screen -The importance of being social -Conflicts on using the screen -Need for support for the screen -The screen at home -Training at the center -Conflicts in the family -Other health issues -Joy in training	-Physical training at home -Physical training at the center -Physical training in general -The difficulties of being a relative to a person with dementia -Social life -Interaction with the screen -Challenges in daily life	**The screen empowers and gives freedom** “It gives me the opportunity to train at home if I don't feel like going out one day. And we each have this personal space, where I am in control of the screen and my wife can do whatever she wants while I am training.” **The screen prevents social life** “I am not so happy with the screen. I feel like I have lost something. Before, when we were training at the center twice a week, it made me so happy. Also, my wife enjoys talking to the other relatives at the center.” **The screen is a friend that structures everyday life** “I get up early and as a first thing I turn to the screen and do some exercises. It is nice to know that it is my own space.” **The screen creates conflicts at home** “It is difficult to motivate him to do the training and I no longer engage in it. Also, I find it irritating that I have to move things around.”	**The screen: a source of increased autonomy or conflicts at home**

## Results

This study aimed to identify barriers and facilitators for the use of MBT in older people with dementia. We identified a permeating theme from the analysis as ‘The screen: a source of increased autonomy or conflicts at home,’ which embraced aspects of potential barriers and facilitators when using MBT as a means for rehabilitation intervention in people with dementia. To maintain anonymity in the subsequent discussion, the participants with dementia are referred to as P1, P2, P3, and P4. Other study participants are referred to by their familial role or professional title.

### The screen: a source of increased autonomy or conflicts at home

When asked about MBT training, participants compared it to training at the center. Instructor and relatives alike reported that participants trained intensely and looked forward to training sessions. Some of the relatives were at first concerned that participants would drop out and reported that in the beginning, they had to motivate participants to leave the house for training:

Well, at first, he was complaining that he had to go, and I think it was general for the participants; the group were all grumpy men. But we, the wives insisted. He has a regular training partner and they are like two little boys at school. They do have good cohesion in the group. They need each other; they need somebody to be with, someone who has similar challenges as themselves. They are already very much alone. I don't think the screen is useful for people with dementia. [Relative]

Entering phase 3, the participants were offered the home-based MBT device (referred to as “the screen”) and group training reduced to once a week. The instructor sensed that reduction of the group sessions had consequences for participants' levels of social activity and that social coherence in the group was disrupted. This observation was shared by relatives and the participants themselves:

What I like about it is training at the centre. We have fun and it gives a kind of boost. But training with the screen – it is not the same. Not at all. At the center it is completely different. I can tell that many enjoy it, and it gives them a boost. I would rather train together in a group. I don't mind the screen, but the socialness we share is something else and we support each other. The other participants say that the screen is a bit difficult; that they are not too happy about it. They are not so straightforward about it, ehh…if they feel that it is silly to train alone in front of a screen [laughs]…It is something like that I think. [P1]

The perspective that participants experienced a loss of social exposure was shared by their relatives. Some relatives expressed that home-based MBT was a good idea, but not a suitable tool for people with dementia. A person with dementia is already socially isolated and more efforts should be made to establish relationships to other persons with dementia:

Well, my husband was upset. He has been so pleased with the group he has been training with. And now the group is split into two, one half training at home and only at the centre once a week. The other group continue to meet twice a week, but with only four participants, and that is too few. [Relative]

Although the participants felt that they had lost something by training at home, two of the participants followed the MBT training program every day and enjoyed it. However, they all agreed that the screen could not replace training in groups.

For the two participants training frequently at home, the MBT sessions became part of their daily routine. Together with relatives, they had installed the screen in a separate and private room with plenty of space to do the exercises without having to move furniture around. The other two participants did not have this option and used the MBT device maybe once a week or less. One had placed it in the bedroom, which meant that he had to move items and close the curtains. Another participant had it placed in the living room next to photos of family members.

The two daily users of the MBT had a routine of starting early in the morning. P1 was an early riser, and his wife gave him domestic tasks that he was able to manage independently. Beforehand she would have prepared recipes and shopped for groceries. But training at home she did not interfere with:

He has done the training by himself after the instructor was here to install it. At first, he couldn't make it work and I had to help him. Then it worked fine and I was watching him doing the program. Since then, he didn't complain about it. [Relative]

P2 was also alone in the house during the morning hours and trained independently without being motivated by his relatives: "I get up early. I have always done that, around six or so. And then I go to the room for doing the exercises." P1 and P2 had both been physically active their whole lives and both had participated in team sports. Being physically active was therefore familiar, although age and health placed some limitations on training.

MBT training increased individuals’ sense of freedom since it gave them the choice to train at home instead of going to the center:

If [P2] is sick or has pain in the legs, then he gets upset that he can't go to the center and then he has tried to do the exercises at home three times a day. He has to move otherwise he grows roots. [Relative]

The MBT instructor at the health care center also explained that to some of the participants, the screen increased their independence. Some relatives even reported that the screen facilitated personal space since they got 20–30 min to themselves without being disturbed or concerned about what the person with dementia was doing.

While two of the participants experienced that MBT supported their independence, the other two participants did not use the screen frequently. P3 had difficulties doing the exercises correctly since he had back problems. As his relative explained:

He easily manages the screen, turning it on and off and so. But the exercises are difficult. They are actually the same as at the center. In the beginning I was always there to support him, then I could explain to lift the right arm and left leg…ehh, he can't tell the difference between left and right, ehh…and then he asks, ‘What should I do now?’ The screen can't help him with that. No, there must be someone to instruct him that he has to bow deeper, to move the head, to stand up straight and so on. Cause if you don't do it right then the screen tells you that you haven't done them yet. [Relative]

P3 himself expressed that he had problems doing the exercises due to his stiff back. He also found it difficult to be motivated and perceived it as a duty more than a pleasure. He did enjoy going to the center, although at times he found it difficult to leave the house.

Training by MBT at home caused conflicts between participants and their relatives. The technology was challenging; participants were not motivated, or the exercises were too difficult. The instructors reported cases where the screen was returned:

Some of the relatives have contacted us to come and pick up the screen since it caused conflicts. There was a case where the participant had the idea that it worked really well for him and he was training each day. But I could see that he didn't. I went to their house to observe him doing the program and I could tell that it worked for him. But he couldn't do it by himself, and he would not let his wife help him. And they had conflicts. Then he said that he trained when she wasn't at home. But he still didn't, according to the screen reports. [Instructor]

One relative experienced several conflicts with the participant due to the screen. During the interview, they disagreed when it came to frequency of usage, the placement of the screen and technical details. The relative believed that it was too slow to start up, that the participant didn't train at all, and that it was a problem to have it placed in the bedroom and having to move things around when using it. The patient disagreed with all these statements, however.

It was not only the screen that caused relational conflicts. Relatives were highly affected by changes in behaviour and personality, and daily routines were changed drastically in some instances, leading to major changes in many relationships. In such cases, training at home could cause more burden than benefit.

## Discussion

Lack of motivation, difficulties with the screen, and difficulties completing the exercises were all experienced as barriers to use of the screen. Additionally, some of these barriers caused challenges in home life, such as disagreements and extra burdens placed on relatives. Irrespective of whether the screen increased independency or conflict, all participants agreed that by training at home, they suffered a social loss. Training in a group was a meaningful activity that provided an opportunity to socialize with likeminded individuals. In general, people with dementia are at higher risk for social isolation. Their ability to participate in social interaction is limited, but nevertheless meaningful [29]. Group training with peers is therefore crucial to prevent social isolation.

Studies that have investigated group training for people with dementia have shown group training to be more effective in improving cognitive functions compared to solo training [16, 30]. Relationships have also been shown to facilitate exercise participation. A study of nursing home residents with dementia found that group exercises were experienced as motivating and that individuals used each other as role models [10]. Similar results were found in a study of older people with dementia participating in high-intensity group exercise [9]. Participants found inspiration in watching each other perform the exercises and competing, and emphasized the importance of being with others in the same situation [9]. The importance of peer interaction was also identified in a recent review study, where it was found that peer interactions supported a sense of belonging [31]. These results were also found in the present study: participants and relatives alike shared the view that group training was meaningful, resulting in laughter, good company, intense training and friendly competition. Based on these results, we encourage the inclusion of group exercises in future physical activity interventions targeting people with dementia in order to support social interactions.

To some of the participants in this study, training at home was a helpful tool in creating a routine they could control themselves in regards to training frequency and intensity. Training at home may in this sense be seen as enhancing a sense of independence. Moser (2011) argues that dementia is articulated in a biomedical framework in which the disease gradually overtakes, undermines and diminishes the "I" [32]. Maintaining self-initiative and independence becomes crucial and possibly even a deliberate choice in order to maintain the "I" [33]. Research has also shown that preserving autonomy can be seen as a self-maintaining strategy [34]. Following this rationale, engaging in MBT training at home may be seen as a symbolic (or literal) act of maintaining autonomy and independence.

Turning to the literature of human interaction with technology, we found inspiration in the concept of *domestication*, which refers to the process by which technology is integrated into daily life and helps explain the processes that people engage in when implementing technological equipment in daily routines at home [35]. Domestication implies the process of connecting the private sphere with a professional sphere and transforming the strange into the familiar. A similar process took place in the current study. The placement of the screen varied in the four different homes visited. For two of the participants, the screen had been given its own room, creating a separate space for training. For these same two individuals, MBT worked well and became part of the daily routine. One explanation may be that having a separate space invited completion of the training as it provided segregation between 'cosy home' and ' training space.' The home, décor, available physical space, and the symbolic meanings that residents attach to 'home' all influence the adaptation of technology in daily life. The concept of domestication has potential for beneficial effects if made explicit in situations when implementing technology like MBT in the home.

Transforming the home into a space for physical activity shifts responsibility for a health-promoting activity. At the local training center, the participants were supported, encouraged and inspired by each other and by the instructor. At home, they relied on their own motivation and on support from relatives. In the specific context of engaging people with dementia in physical activity, we argue that this practice is part of the wider policy discourse on active ageing. The growing ageing population has made it necessary for policy makers to rethink healthcare systems and behavioral interventions to maintain older persons’ independence and thus delay age-related health problems for as long as possible [36]. In this discourse, independence is enhanced as the key to quality of life in old age [37]. However, as has been discussed in the work of Lupton, governmental health campaigns aimed at informing citizens about how to maintain health through individual behavior may primarily result in behavioral changes by individuals who are already primed in terms of motivation or resources. The consequences may be that vulnerable population groups—those who lack either motivation or resources or other key factors—are not able to engage in health-maintaining activities, potentially resulting in further marginalization, especially considering the increasing use of health technologies [38, 39]. In the current study, participants and their relatives differed in the way they trained using the screen at home. It is therefore important to distinguish between the different needs and resources available to each person.

### Strength and weaknesses

The strength of this study lies in the methodological ethnographic design, providing a unique opportunity to observe immediate phenomena during interactions between participants and between participant and technology. This methodological strength was further reinforced during the interviews, as validation was sought via repeating the researcher's interpretation of statements to participants and relatives, allowing for real-time correction as needed [27].

Protection of persons with dementia due to their vulnerability and perceived lack of capacity to give consent has frequently resulted in the exclusion of individuals with dementia from research [40]. Until the 1990s, attitudes towards persons with dementia were that their perceptions were difficult to assess and non-reliable [41]. More recently, however, it has been acknowledged that exclusion of a population group from research raises ethical concerns, since the subjective perspective and experience of these individuals goes unvoiced. Research inclusion contributes to participants feeling useful and provides researchers with important information in regards to the needs, desires and preferences of individuals with dementia [27]. Although research involving people with dementia may present challenges due to possible diminished abilities in communication and abstraction, it is crucial to include the voices of this vulnerable group, to understand their needs, experiences and need for resources [42].

The number of participants in this study was limited: one weakness may be that we were unable to include additional participants. To some extent, the results illustrate divergent perspectives that reflect relevant nuances in using MBT, but the results also call for further studies. The study is part of a feasibility study and points to the need for attention to processes of interaction and subjective experiences in future research.

All the participants with dementia were men. This may be due to the fact that all relatives were female, and it is likely that participation was initiated by the female relatives. Future studies may benefit from women's perspectives on home-based MBT and the support they receive from male relatives. The study did not include persons with severe dementia, and did not study the participants for a longer period. Future studies among individuals with dementia may also consider focusing on changes in the frequency and quality of home-based MBT training as the disease progresses.

## Conclusion

This study explored how MBT is experienced by people with dementia and their relatives participating in an intervention facilitated by a Danish municipality. The study identified potential benefits and challenges resulting from home-based MBT training: the sense of having lost the opportunity to be with like-minded individuals in group training; the screen caused marital conflicts; and the screen enhanced feelings of freedom and independence. There is therefore no clear answer to whether MBT increases quality of life, but it can be seen as a supplement to in-person group training.

## Supplementary Information

**Additional file 1. **Interview guides.

## Data Availability

The datasets generated and/or analysed during the current study are not publicly available due to compliance with DDPA rules on protection of personal data, but are available from the corresponding author on reasonable request.

## Abbreviations

EQOL5: European Quality of Life 5 dimensions questionnaire
ICT: Interactive communication technology
MBT: Motion-based technology
MMSE: Mini-mental state examination
NPI-Q: Neuropsychiatric Inventory Questionnaire
STC: Systematic text condensation

## References

[1] Forbes D, Thiessen EJ, Blake CM, Forbes SC, Forbes S. Exercise programs for people with dementia. Cochrane Database Syst Rev. 2013(12):CD006489. 10.1002/14651858.CD006489.pub3. PMID: 24302466. 10.1002/14651858.CD006489.pub3 24302466

[2] Duggleby WD, Swindle J, Peacock S, Ghosh S (2011). A mixed methods study of hope, transitions, and quality of life in family caregivers of persons with Alzheimer's disease. BMC Geriatr.

[3] van Gennip IE, Pasman HR, Oosterveld-Vlug MG, Willems DL, Onwuteaka-Philipsen BD (2016). How dementia affects personal dignity: a qualitative study on the perspective of individuals with mild to moderate dementia. J Gerontol B Psychol Sci Soc Sci.

[4] Brett L, Traynor V, Stapley P (2016). Effects of physical exercise on health and well-being of individuals living with a dementia in nursing homes: a systematic review. J Am Med Dir Assoc.

[5] Ohman H, Savikko N, Strandberg TE, Pitkala KH (2014). Effect of physical exercise on cognitive performance in older adults with mild cognitive impairment or dementia: a systematic review. Dement Geriatr Cogn Disord.

[6] Sampaio A, Marques EA, Mota J, Carvalho J (2019). Effects of a multicomponent exercise program in institutionalized elders with Alzheimer's disease. Dementia (London).

[7] Sobol NA, Hoffmann K, Frederiksen KS, Vogel A, Vestergaard K, Braendgaard H (2016). Effect of aerobic exercise on physical performance in patients with Alzheimer's disease. Alzheimers Dement.

[8] van Alphen HJ, Hortobagyi T, van Heuvelen MJ (2016). Barriers, motivators, and facilitators of physical activity in dementia patients: a systematic review. Arch Gerontol Geriatr.

[9] Lindelof N, Lundin-Olsson L, Skelton DA, Lundman B, Rosendahl E (2017). Experiences of older people with dementia participating in a high-intensity functional exercise program in nursing homes: "While it's tough, it's useful". PLoS ONE.

[10] Olsen CF, Telenius EW, Engedal K, Bergland A (2015). Increased self-efficacy: the experience of high-intensity exercise of nursing home residents with dementia - a qualitative study. BMC Health Serv Res.

[11] Cedervall Y, Torres S, Aberg AC (2015). Maintaining well-being and selfhood through physical activity: experiences of people with mild Alzheimer's disease. Aging Ment Health.

[12] Prick AE, de Lange J, Scherder E, Twisk J, Pot AM (2016). The effects of a multicomponent dyadic intervention on the mood, behavior, and physical health of people with dementia: a randomized controlled trial. Clin Interv Aging.

[13] Wesson J, Clemson L, Brodaty H, Lord S, Taylor M, Gitlin L (2013). A feasibility study and pilot randomised trial of a tailored prevention program to reduce falls in older people with mild dementia. BMC Geriatr.

[14] Bongartz M, Kiss R, Ullrich P, Eckert T, Bauer J, Hauer K (2017). Development of a home-based training program for post-ward geriatric rehabilitation patients with cognitive impairment: study protocol of a randomized-controlled trail. BMC Geriatr.

[15] Dove E, Astell AJ. The Use of Motion-Based Technology for People Living With Dementia or Mild Cognitive Impairment: A Literature Review. J Med Internet Res. 2017;19(1):e3. 10.2196/jmir.6518. 10.2196/jmir.6518 PMC5266826 28077346

[16] Tanaka S, Honda S, Nakano H, Sato Y, Araya K, Yamaguchi H (2017). Comparison between group and personal rehabilitation for dementia in a geriatric health service facility: single-blinded randomized controlled study. Psychogeriatrics.

[17] Danish Government: Styrket indsats for den ældre medicinske patient [Strengthening health care for the elderly patient]. 2016. [Available from: https://www.sum.dk/~/media/Filer%20-%20Publikationer_i_pdf/2016/Styrket-indsats-for-den-aeldre-medicinske-patient/National_Handlingsplan.pdf.

[18] Boeckxstaens P, De Graaf P (2011). Primary care and care for older persons: position paper of the European forum for primary care. Qual Prim Care.

[19] Williams A (2002). Changing geographies of care: employing the concept of therapeutic landscapes as a framework in examining home space. Soc Sci Med.

[20] Wilson DM, Hewitt JA, Thomas RE, Woytowich B (2014). Why did an out-of-hospital sift of death and dying occur in Canada after 1994?. Int J Palliative Care.

[21] Danish Government: Demenshandlingsplan 2025 [National plan for Dementia] 2016 [Available from: https://www.sum.dk/Aktuelt/Publikationer/~/media/Filer%20-%20Publikationer_i_pdf/2016/Demenshandlingsplan-2025-PUB-sept-2016/Handlingsplan-V2.ashx.

[22] Astell AJ, Czarnuch S, Dove E. System Development Guidelines From a Review of Motion-Based Technology for People With Dementia or MCI. Front Psychiatry. 2018;9:189. 10.3389/fpsyt.2018.00189. 10.3389/fpsyt.2018.00189 PMC5968379 29867610

[23] Littbrand H, Lundin-Olsson L, Gustafson Y, Rosendahl E (2009). The effect of a high-intensity functional exercise program on activities of daily living: a randomized controlled trial in residential care facilities. J Am Geriatr Soc.

[24] Petersen JD, Larsen EL, la Cour K, von Bülow C, Skouboe M, Christensen JR, Waldorff FB. Motion-Based Technology for People With Dementia Training at Home: Three-Phase Pilot Study Assessing Feasibility and Efficacy. JMIR Ment Health. 2020;7(8):e19495. 10.2196/19495. 10.2196/19495 PMC7481868 32845243

[25] Hellstrom I, Nolan M, Nordenfelt L, Lundh U (2007). Ethical and methodological issues in interviewing persons with dementia. Nurs Ethics.

[26] Davies CA (2008). Reflexive ethnography: a guide to researching selves and others.

[27] Beuscher L, Grando VT (2009). Challenges in conducting qualitative research with individuals with dementia. Res Gerontol Nurs.

[28] Malterud K (2012). Systematic text condensation: a strategy for qualitative analysis. Scand J Public Health.

[29] Phinney A, Chaudhury H, O'Connor DL (2007). Doing as much as I can do: the meaning of activity for people with dementia. Aging Ment Health.

[30] Junge T, Ahler J, Knudsen HK, Kristensen HK (2020). The effect and importance of physical activity on behavioural and psychological symptoms in people with dementia: a systematic mixed studies review. Dementia (London).

[31] Junge T, Knudsen HK, Kristensen HK (2020). The effect of long-term, group-based physical, cognitive and social activities on physical performance in elderly, community-dwelling people with mild to moderate dementia. Dementia.

[32] Moser I (2011). Dementia and the limits to life: anthropological sensibilities, STS interferences, and possibilities for action in care. Sci Technol Hum Val.

[33] MacQuarrie CR (2005). Experiences in early stage Alzheimer's disease: understanding the paradox of acceptance and denial. Aging Ment Health.

[34] Clare L (2003). Managing threats to self: awareness in early stage Alzheimer's disease. Soc Sci Med.

[35] Lie M, Sørensen KH (1996). Making technology our own?: Domesticating technology into everyday life.

[36] Zaidi A, Howse K (2017). The policy discourse of active ageing: some reflections. J Popul Ageing.

[37] Kamp A, Hvid H (2012). Elderly care in transition - Management, meaning and identity at work. A scandinavia perspective.

[38] Lupton D. The imperative of health: Public health and the regulated body. SAGE Publications Ltd; 1995. 10.4135/9781446221976.

[39] Lupton D (2018). Digital health.

[40] Witham G, Beddow A, Haigh C (2015). Reflections on access: too vulnerable to research?. J Res Nurs.

[41] Murphy K, Jordan F, Hunter A, Cooney A, Casey D (2015). Articulating the strategies for maximising the inclusion of people with dementia in qualitative research studies. Dementia (London).

[42] Phillipson L, Hammond A. More than talking: a scoping review of innovative approaches to qualitative research involving people with dementia. Int J Qual Meth. 2018;17(1). 10.1177/1609406918782784.

[43] National Committee on Health Research Ethics. Act on research ethics review of health research projects. 2020 [Available from: https://en.nvk.dk/rules-and-guidelines/act-on-research-ethics-review-of-health-research-projects.

[44] West E, Stuckelberger A, Pautex S, Staaks J, Gysels M (2017). Operationalising ethical challenges in dementia research-a systematic review of current evidence. Age Ageing.

