# Supplementary material for: Home-based training technology for persons with dementia: a qualitative study of barriers and facilitators for mobility-based training at home
Source: BMC Geriatr. 2022 Oct 14;22:800. doi: 10.1186/s12877-022-03505-6 (PMC9569075; doi:10.1186/s12877-022-03505-6)
Supplement: Supplementary file 1 — Additional file 1. Interview guides. [file 12877_2022_3505_MOESM1_ESM.docx]

### Interview Guides

| **Participant** | **Topic and description of the content** |
| --- | --- |
| **Project worker** | **Training at the center** *dialogue concerning training at the center: frequency, type of exercises, how much support is needed for performing the exercises, relations between the participants and relations between participants and instructor.* |
|  | **MBT Training at home** *dialogue concerning installation of MBT at home: If and how MBT matches the physical and cognitive level of participants, the placement of MBT, frequency and type of exercises, type of support needed: motivation, technology, exercises. Gain and loss concerning training at home.* |
|  | **Person with dementia** *dialogue concerning participants with dementia, their characteristics, type of dementia, participant who are easy versus difficult to motivate, the typical participant versus the non-typical participant, familiarity with technology and previous training.* |
|  | **Relatives** *dialogue concerning relatives: their motivation for being engaged in the project, their support to the person with dementia, their needs as a relative to a person with dementia.* |
| **Person with dementia** | **Background information** *dialogue concerning age, previous occupation, family, education.* |
|  | **Training at the center** *dialogue concerning training at the center frequency, type of exercises, how much support is needed for performing the exercises, motivation for participation, relations between the participants and relations between participants and instructor* |
|  | **MBT training at home** *dialogue concerning installation of MBT at home: If and how MBT matches the physical and cognitive level of participants, the placement of MBT, frequency and type of exercises, type of support needed: motivation, technology, exercises. Gain and loss concerning training at home* |
|  | **Experiences with physical activity** *dialogue concerning physical activities in earlier years: type, frequency, gains.* |
|  | **Experiences with technology** *dialogue concerning the use of technology in earlier years: PC work, smart phone, Ipad.* |
| **Relative** | **Background information** *dialogue concerning age, previous occupation, family, education.* |
|  | **Motivation for participating in the project** *dialogue concerning reasons for engaging in the project.* |
|  | **Benefits and challenges for the person with dementia** *dialogue concerning benefits and challenges for the person with dementia participating in training at the center and at home.* |
|  | **Benefits and challenges for you as a relative** *dialogue concerning needs of support as a relative to a person with dementia. The type and frequency of support given to the person with dementia in general and in relation to physical training at the center and at home.* |
